# Combining immigration records with a postpartum population-based survey to assess prevalence of perinatal psychosocial and behavioral risk factors among immigrant subgroups.

**DOI:** 10.23889/ijpds.v7i3.1853

**Published:** 2022-08-25

**Authors:** Roheema Ewesesan, Mariette Chartier, Nathan Nickel, Elizabeth Wall-Wieler, Marcelo Urquia

**Affiliations:** 1 University of Manitoba

## Objectives

Perinatal risk factors can vary by immigration status. To advance knowledge on sociobehavioral health risks among pregnant and childbearing immigrant women, we compared perinatal health indicators between immigrant and non-immigrants, overall, and according to key immigrant characteristics (refugee status, secondary migration, birth region, and duration of residence).

## Approach

We conducted a population-based cross-sectional study of 33,754 immigrant and 172,342 non-immigrant childbearing women in Manitoba, Canada, aged 15-55 years, who had newborn screening data completed by public health nurses within two weeks postpartum from 2000 to 2017. The screening data was linked to a Canadian national immigration database. Additional databases were linked to collect demographic and perinatal clinical information. Logistic regression models were used to examine the associations between immigration characteristics and perinatal health indicators, such as social isolation, relationship distress, partner violence, depression, alcohol, smoking, substance use and late prenatal care initiation.

## Results

More immigrant women reported being socially isolated (12.3%) than non-immigrants (3.0%) (Adjusted Odds Ratio (aOR): 6.90, 95% Confidence Interval (CI): 6.53, 7.28) but exhibited lower odds of other outcomes. In the analysis restricted to immigrants, recent immigrants (< 5 years of stay) had higher odds of being socially isolated (aOR: 9.29, 95% CI: 7.80, 11.06) and late prenatal care (aOR: 1.73, 95% CI: 1.23, 2.42) compared to long-term immigrants, but lower odds relationship distress, depression, alcohol, smoking and substance use. Refugee status was positively associated with social isolation, relationship distress, depression, and late prenatal care whereas secondary migration was protective for social isolation, relationship distress, and smoking. Relationship distress and behavioral health indicators varied by maternal birth region.

## Conclusion

The novel linkage of birth screening data with the immigration data advances knowledge on immigrant perinatal health by identifying risk patterns for multiple psychosocial and behavioral health indicators, highlighting subgroups at higher and lower risk of exposures that may contribute to adverse perinatal health outcomes.

